# Independent Evaluation of the Respective Predictive Values for High-Grade Prostate Cancer of Clinical Information and RNA Biomarkers after Upfront MRI and Image-Guided Biopsies

**DOI:** 10.3390/cancers12020285

**Published:** 2020-01-24

**Authors:** Mathieu Roumiguié, Guillaume Ploussard, Léonor Nogueira, Eric Bruguière, Olivier Meyrignac, Marine Lesourd, Sarah Péricart, Bernard Malavaud

**Affiliations:** 1 Department of Urology, Institut Universitaire du Cancer, 31059 Toulouse, France; marine_lsrde@hotmail.fr (M.L.); bernard.malavaud@me.com (B.M.); 2 Department of Urology, La Croix du Sud, 31130 Quint Fonsegrives, France; g.ploussard@gmail.com; 3 Institute of Biology, CHU Purpan, 31059 Toulouse, France; nogueira.l@chu-toulouse.fr; 4 Department of Radiology, Clinique Pasteur, 31076 Toulouse, France; bruguiere.eric@gmail.com; 5 Department of Radiology, Institut Universitaire du Cancer, 31100 Toulouse, France; meyrignac.olivier@iuct-oncopole.fr (O.M.); pericart.sarah@iuct-oncopole.fr (S.P.)

**Keywords:** multi-parametric MRI, prostate cancer, diagnosis, targeted biopsy, biomarkers

## Abstract

Upfront MRI is taking the lead in the diagnosis of clinically significant prostate cancer, while few image-guided biopsies (IGBs) fail to demonstrate clinically significant prostate cancer. The added value of innovative biomarkers is not confirmed in this context. We analysed SelectMDx-v2 (MDx-2) in a cohort of upfront MRI and image-guided biopsy patients. Participants included patients who received a trans-rectal elastic-fusion registration IGB on the basis of DRE, PSA, PCA3, and PCPT-2.0 risk evaluation. Pre-biopsy MRI DICOM archives were reviewed according to PI-RADS-v2. Post-massage first-void urine samples stored in the institutional registered bio-repository were commercially addressed to MDxHealth to obtain MDx-2 scores. Univariate and multivariate analyses were conducted with the detection on IGB of high-grade (ISUP 2 and higher) as the dependent variable. High-grade cancer was demonstrated in 32/117 (27.4%) patients (8/2010–8/2018). Age, prostate volume, biopsy history, MDx-2, and PI-RADS-v2 scores significantly related to the detection of high-grade cancer. MDx-2 scores and the clinical variables embedded into MDx-2 scores were analysed in multivariate analysis to complement PI-RADS-v2 scores. The two combinations outperformed PI-RADS-v2 alone (AUC-ROC 0.67 vs. 0.73 and 0.80, respectively, *p* < 0.05) and calibration curves confirmed an adequate prediction. Similar discrimination (C-statistics, *p* = 0.22) was observed in the prediction of high-grade cancer, thereby questioning the respective inputs and added values of biomarkers and clinical predictors in MDx-2 scores. Based on the results of this study, we can conclude that instruments of prediction developed for systematic prostate biopsies, including those that incorporate innovative biomarkers, must be reassessed and eventually confirmed in the context of upfront MRI and IGB.

## 1. Introduction

Current efforts in prostate cancer diagnosis follow a fine line between evidencing significant cancers that warrant intervention and controlling the overdiagnosis of insignificant lesions. Toward that end, urologists rely on three dimensions—clinical, biological, and radiological—used independently, in conjunction, or sequentially. A recent modelling of diagnostic avenues [1] based on the PROMIS cohort supported upfront MRI as a dominant strategy in biopsy-naive patients to inform the suspicion and location of putative lesions [2], although it might substantially reorient medical resources and expenditures. Surprisingly, to date, no efforts have been made to introduce predictive tools [3] or innovative biomarkers [4] in the process of selection, although they have been heavily researched and promoted in the precedent strategy of clinically driven systematic ultrasound-guided biopsies.

The highly competitive market of biomarker research has followed different avenues, such as PSA trafficking (Prostate Health Index, 4K score) and cancer-specific non-coding RNAs (PCA3) eventually combined with fusion genes (MIMPS), and has more recently acknowledged the added value of clinical information (SelectMDx-v2 (MDx-2), STHLM3) [5]. 

MDx-2, which belongs to the latest generation of biomarker-based models of prediction, was selected for this study as it could be retrospectively tested from archived prostate massage samples. This proprietary model of prediction combines biomarker expressions in prostatic epithelium with clinical risk factors and was recently mentioned in the EAU guidelines to avoid unnecessary biopsies. Hendriks reported a strong correlation with MRIs secondarily performed for persistent clinical suspicion or for cancer staging—a double-edged result as it simultaneously vindicated MDx-2 as a proxy for significant cancer that constitutes the bulk of PI-RADS scores 4 and 5, but also questioned the value that it might have retained in the reverse situation of upfront MRI [6]. 

Here, we took advantage of a registered collection from patients who received MRI prior to software-assisted image-guided biopsies to independently evaluate the predictive performances of SelectMDx-v2—a recently updated model that computes clinical data and innovative biomarkers to estimate the odds of high-risk cancer on systematic biopsy [7]. 

## 2. Results

### 2.1. Patient Characteristics

**Figure 1:**
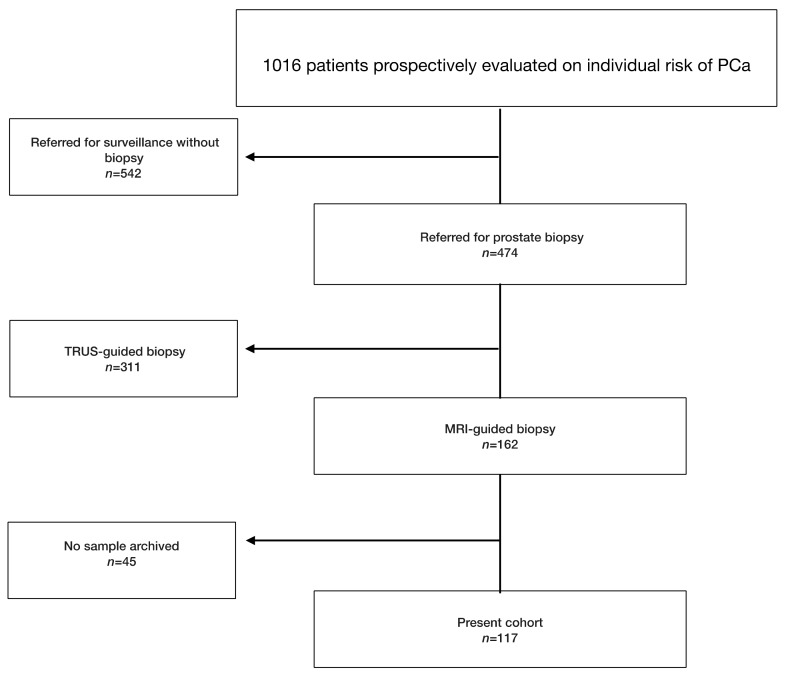
Flowchart presenting the selected study population. PCa: prostate cancer.

Of 1016 patients, 117 (11.5%) received image-guided biopsy (IGB) after MRI (Figure 1). Patient characteristics and biopsy results are presented in Table 1. Systematic cores detected two additional significant cancers in biopsy-naive patients and none in repeat-biopsy patients.

### 2.2. Prediction of the Biopsy Outcomes

**Figure 2:**
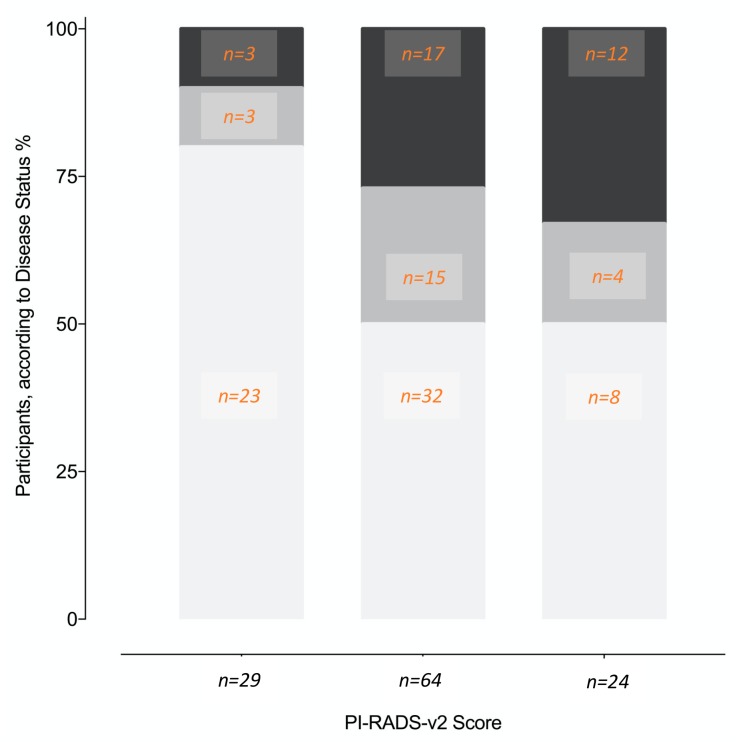
Proportions of different disease status (%, number of patients) on image-guided biopsy according to the PI-RADS-v2 score lesions. No cancer (white), non-high-grade cancer (light grey), high-grade cancer (dark grey).

**Figure 3:**
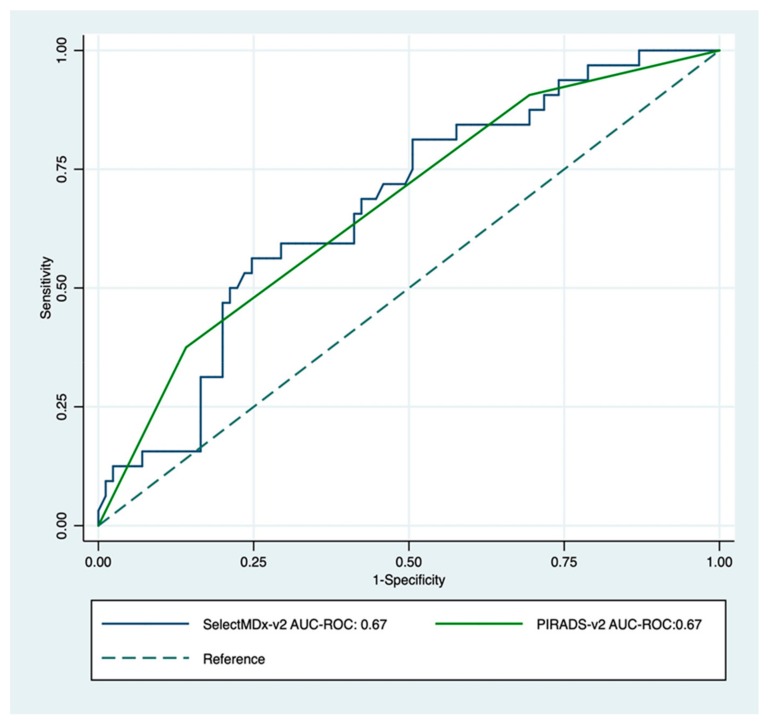
Receiver operating characteristic curve for PI-RADS-v2 score (green) and SelectMDx-v2; (blue) for the detection of high-grade cancer on image-guided biopsy. The dotted diagonal line is the reference line (AUC = 0.5).

Age, prostate volume, biopsy history, MDx-2, and PI-RADS-v2 scores—but not PCPT-2.0 scores—significantly related to the detection of high-grade cancer (Table 1). As expected, PI-RADS-v2 scores were strongly related to biopsy results (Figure 2). The AUC-ROC showed similar discrimination between MDx-2 and PI-RADS-v2 scores (0.67 and 0.67, *p* = 0.99, respectively) (Figure 3).

**Figure 4:**
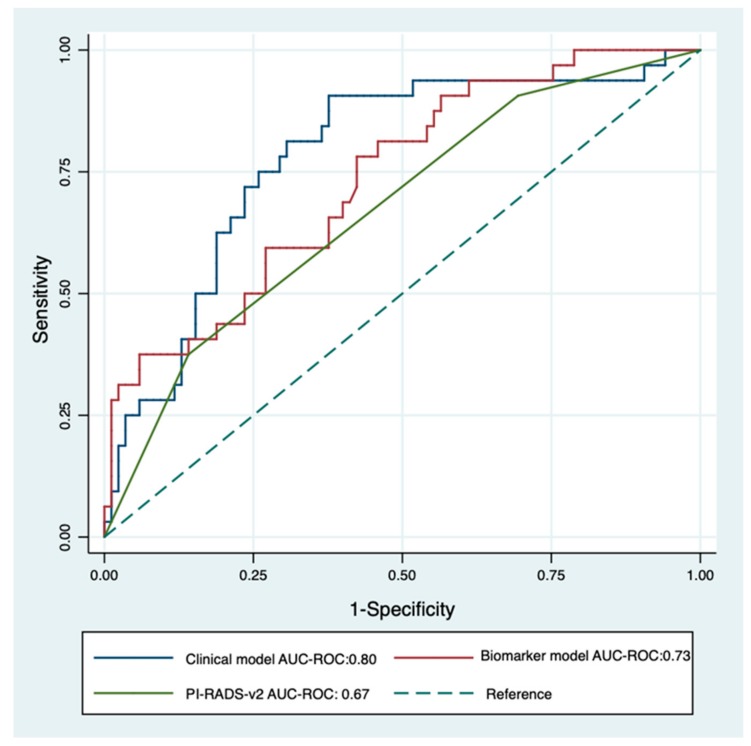
Receiver operating characteristics curve for PI-RADS-v2 score (green) and multivariable logistic regression of clinical model (PI-RADS-v2 score + PSA density + Age + DRE; blue) and biomarker model (PI-RADS-v2 score + SelectMDx-v2; red) for the detection of high-grade cancer on image-guided biopsy. The dotted diagonal line is the reference line (AUC = 0.5).

**Figure 5:**
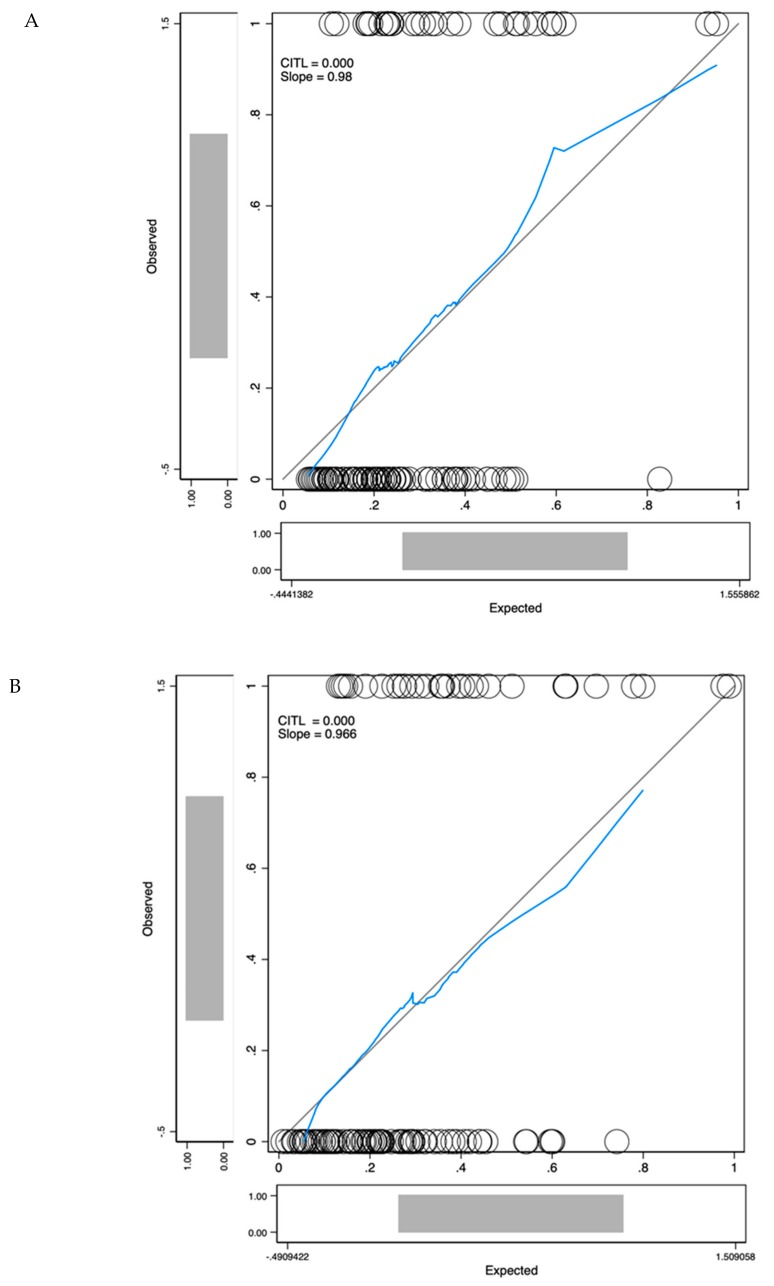
Calibration curves for the biomarker model (**A**) and the clinical model (**B**). The *x*-axis represents model predictions, the *y*-axis the observed diagnosis of high-grade cancer. Calibration in the large (CITL) measures whether the predicted prevalence was less than (CITL < 0) or greater than (CITL > 0) the observed prevalence.

MDx-2 scores are modelled from clinical variables and mRNA expression levels. To prevent redundancy, two separate multivariate analyses were conducted complementing PI-RADS-v2 scores with either MDx-2 scores (biomarker model) or with the sole clinical variables embedded into the MDx-2 score (clinical model). Both models (Table 2) outperformed PI-RADS-v2 (AUC-ROC 0.67 vs. 0.73 and 0.80 for *biomarker* (*p* < 0.05) and *clinical* models (*p* = 0.013), respectively, Figure 4) and showed similar discrimination (C-statistics, *p* = 0.22). The proportion of patients correctly classified with high-grade cancer ranged from 47.0% (PI-RADS-v2) to 73.7% (clinical model, Table 3). Calibration curves confirmed adequate prediction for both models (Figure 5A,B).

## 3. Discussion

Imaging was recently vindicated in the EAU guidelines that introduced upfront mpMRI in the detection of prostate cancer [8], although accessibility, inter-observer variability and the grey zone of intermediate PI-RADS-v2 score 3 findings [9] may hamper its diffusion [10]. Washino recently observed that no PI-RADS-v2 score 3 lesions proved positive on transperineal systematic or cognitive biopsies when PSA density was lower than 0.15, suggesting that biology could help refine the risk estimation based on MRI [11]. Similarly, the EAU guidelines also promoted the use of risk calculators or biomarkers to contain unnecessary biopsies in patients with intermediate PSA values, although none of the available instruments that were developed in the obsolete context of systematic biopsies received independent validation in the emerging era of upfront MRI and IGB. 

Diagnostic performances are highly dependent on the perception error, which is the accuracy of the technique used to demonstrate the presence of cancer [12]. It is therefore anticipated that by controlling the risk of false-negative biopsies—otherwise understood as false-positive results of the test—upfront MRI and IGB would improve the diagnostic performances of risk calculators or biomarkers. Here, on the contrary, the recommended cut-off yielded lower sensitivity and specificity (84.4% and 35.3%, respectively) than reported in the seminal MDx-2 report (96% and 53%, respectively) [7] and correctly classified only one patient out of two (48.7%, Table 3). Adjusting the cut-off value by balancing the sensitivity and the specificity of the MDx-2 model with the Youden index increased this proportion to 70.1% (Table 3). The magnitude of this effect highlighted that models developed based on historical series of random systematic biopsies could not be transferred to the situation of upfront MRI and IGB without being reconfirmed. 

Models of prediction are also highly dependent on the underlying characteristics of the population and the disease prevalence, and it is unclear how the process of selection may affect the performance of biomarkers [12]. In the PRECISION study, upfront MRI and IGB failed to demonstrate high-grade cancer in approximately half of the patients (86/181, 47.5%), highlighting the need of instruments capable of further refining the indication of IGB after imaging. In our centre, where patients received image-guided biopsies only after standardized clinical and biological evaluation, PSA, PSA density and PCA3 values that constituted the first step in the process of selection no longer informed the outcome of image-guided biopsies (Table 1). It was therefore of high interest to research whether new players in the field of biomarkers might highlight some relevant information that could constitute a third layer of decision after routine clinical indicators (PSA, prostate volume) and MRI. 

Here, we confirmed that MDx-2 still carried independent information on high-grade cancer (Table 2) and observed on ROC curve analysis that it significantly improved the discrimination of PI-RADS-v2 (AUC-ROC PI-RADS-v2: 0.67 vs AUC-ROC biomarker model: 0.73, *p* = 0.04, Figure 4). The corresponding calibration curve (Figure 4) further confirmed the value and the accuracy of complementing MRI information with MDx-2 scores, thereby consolidating the hypothesis of positioning MDx-2 after MRI evaluation in the modern pathway of image-based diagnostics. Since the seminal report by Van Neste [7], MDx-2 scores are computed from clinical variables and from the expression levels of two up-regulated genes, homeobox C6 *(HOXC6)* [13] and distal-less homeobox 1 *(DLX1)*—a regulator of the transcription of members of the TGFb superfamily [14]. The respective inputs of the clinical variables and the biomarkers in the prediction were recently adapted after the first return of experience [15], although the model is proprietary and the users are only informed of arbitrary score units that relate to the presence of either cancer or high-grade cancer. Haese recently reported in systematic sextant biopsies patients that *HOXC6* and *DLX1* measurements improved the calibration of the model, compared to the clinical risk factors alone. The net benefit of the biomarkers was mainly observed for low-risk situations [15]—a condition shown by Hendricks to be most prevalent in low PI-RADS scores [6]. However, in this series, only a minority was imaged prior to biopsies and none received IGB, thereby questioning the practical value of this observation. 

Here, in the modern setting of upfront MRI and IGB, MDx-2 scores readily complemented PI-RADS-v2 information and as shown in the calibration curve (Figure 5A), allowed optimal classification in the low-risk segment that was only comprised of PI-RADS score 3 (Figure 2). We conclude that predictive instruments based on MDx-2 could be invaluable to evaluate the benefit of IGB of PI-RADS-v2 score 3 abnormalities, which are quite frequent and were rarely positive (12%) for high-grade cancer in the pivotal PRECISION study [9]. 

As previously emphasized, MDx-2 was constructed as a predictive model rather than a biological test. In the present commercial setting, we were informed of MDx-2 scores but not of *HOXC6* and *DLX1* mRNA expression levels and could not comment on their relevance when isolated. 

On the contrary, the clinical factors included in the MDx-2 model were available in all patients, spurring us to research whether they complemented MRI in the discrimination of high-risk cancer. This integrative approach based on MRI and clinical variables was recently defended by the ERSPC, although in the select subset of biopsy-naive patients evaluated with systematic transperineal grid biopsies [16]. Surprisingly, ROC curve analysis and calibration curve showed that clinical factors (age, serum PSA, prostate volume, DRE) improved discrimination (AUC-ROC PI-RADS-v2: 0.67 vs. AUC-ROC clinical model: 0.80, *p* = 0.006, Figure 4) and classification (Figure 5B) in magnitudes similar to MDx-2 scores. Consistent with the concept of lean medicine [17], the present report confirmed that, aside from the heavily promoted market of biomarkers, relevant complementary information can be extracted from classical, readily available and inexpensive clinical variables.

Indeed, age and prostate volume proved to be robust indicators of high-grade cancer on image-guided biopsies. Prostate volume carried a negative coefficient in the clinical model, attesting that the proportion of high-grade cancers on image-guided biopsies declined with higher prostate volumes, possibly in relation with PSA density or precision of targeting. Within the limited range of PSA in the present series, higher prostate volumes readily translated into lower PSA density (*r* = −0.31, *p* < 0.001)—a robust indicator of healthy prostates [18]. Conversely, fewer positive biopsies might also reveal a higher rate of false-negative results in larger prostates. We recently reported that irrespective of the position of the target from the apex to the base of the prostate, the mean precision of transrectal elastic fusion registration was in the millimetre range [19]. In a recent series of reclassification biopsies, the index cancer was better characterized by transperineal IGB than by the initial transrectal IGB, which highlighted that even when informed by MRI and assisted by advanced software technology the transrectal approach retained some degree of imprecision [20]. This may increase with the size of the prostate, as for geometric reasons, the imprecision driven by minute imperfections in the positioning of the needle augments along the distance between the tip of the needle and the target, which is homogenous to the size of the prostate for targets not strictly located in the posterior aspect of the peripheral zone. 

Several limitations should be considered. Firstly, PI-RADS-v2 and MDx-2 were obtained from archived information (DICOM archives, frozen samples), that is, after the biopsies, although all clinical data were prospectively accrued. Secondly, the decision to recommend MRI and IGB was left to the referring physicians, informed by PCPT-v2 risk estimates, PCA3 scores, PSA serum values, and PSA densities. The present cohort was therefore selected from the general population, although PCPT 2.0 estimates of high-grade cancer (28%, Table 1) and the biopsy outcomes in relation to PI-RADS-v2 scores (Figure 2) suggested a mild-risk population, comparable to the PRECISION study population [9]. Lastly, in the absence of external validation, this report should be considered as preliminary. 

## 4. Materials and Methods

From 2010, PSA and PCA3 testing were offered to patients referred with a clinical suspicion of cancer on the basis of PSA and DRE. Prostate massage (three strokes per lobe) followed PCA3 users’ manual, and 20 mL of the first void mixed with PCA3 buffer to prevent degradation of mRNA were stored at –80 °C in a centralized bio-repository (#DC-2010-1142). Age, 5-ARI, family history, previous biopsy, and TRUS-measured prostate volume (ellipsoid formula) that were necessary to compute PCPT-2.0 scores [21] were recorded. Referring physicians were informed of PSA and PCA3 results as well as of PCPT-2.0 risk estimates (any cancer and Gleason Score ≥7 cancer). Further management was left to the physicians’ discretion (Figure 1) and recorded (MRI results, biopsy technology, and core-by-core biopsy results).

### 4.1. Study Population

The study population consisted of all patients who received (8/2010–8/2018) image-guided biopsy (IGB) by elastic fusion registration (Urostation, Koelis, Cambridge, MA, USA) after risk evaluation. IRB approval was waived in view of the informed consent form signed for biobanking and of the observational nature of this research. Cores were characterized according to the technique used to procure them (image-guided or random systematic) and pathology. 

### 4.2. SelectMDx-v2 Testing

Frozen first-void urine samples archived at the time of PCA3 evaluation were transferred to MDxHealth (MDxHealth, Nijmegen, The Netherlands) to obtain patients’ MDx-2 scores. MDxHealth collaborated with the investigators as a commercial contractor and was blinded to the final outcome of biopsies. The cut-off value of –2.8 proposed by MDxHealth [7,15] was used to obtain the sensitivity and specificity of the test in the detection of high-grade prostate cancer on biopsy.

### 4.3. Magnetic Resonance Imaging

MRI sequences followed the PI-RADS-v2 guidelines [22] and the European consensus meeting on MRI [23]. Fast spin-echo T2-weighted (T2W) images were first obtained. Functional sequences were acquired in the same plane as that of T2W. Diffusion-weighted MRI (DWI) included diffusion-weighted images and the apparent diffusion coefficient map. Dynamic contrast-enhanced MRI was performed with a fat-saturated T1-weighted gradient-echo sequence after injection of gadolinium chelate. Following ESUR recommendations, sequences were first analysed independently [22] and the five-point PI-RADS-v2 scale used to stratify the degree of suspicion [24]. MRI DICOM archives were reassessed by a single experienced uroradiologist (EB) according to the PI-RADS-v2 recommendations [24]. The index target was defined as the largest PI-RADS ≥ 3 volume.

### 4.4. Image-Guided Biopsies

An FDA-approved MRI/3D-TRUS fusion-guided Koelis system that combines a 3D-TRUS unit and a proprietary workstation was used to load and display in 3D the axial T2W and DWI images [19,25]. Axial sections were contoured to obtain the reference MRI prostate volume and to model the target. The reference TRUS volume was then obtained by means of a motorized transrectal probe (HD9, Philips-Healthcare, Best, Netherlands). At a final step, the workstation produced elastic registration of MRI and TRUS reference volumes, which allowed the target to be displayed within either TRUS or MRI reference volumes. Three-to-four fusion IGBs were taken and referenced to MRI and 3D-TRUS archives and processed by uropathologists to document on a core-by-core basis the presence and length of cancer, as well as the primary and secondary Gleason scores. Pending the definition of predictors of significant cancer for lesions detected by MRI-US fusion targeted biopsy [26] adverse pathological features on a core were defined as ISUP 2 or higher. Two additional systematic biopsies (SBs) were obtained in the peripheral zone of the sextants not addressed by IGB. 

### 4.5. Statistical Analysis

A univariate analysis tested the relationships between patients’ characteristics, SelectMDx-v2, PCPT-2.0, and PI-RADS-v2 scores and diagnostic of high-grade cancer. Mann–Whitney U test was used for continuous variables; the Yates’ corrected chi-square test was used for categorical variables. Logistic regression assessed the independence of clinical factors and MDx-2 scores and was used to model predictive instruments; the C-Statistics compared the corresponding ROC curves. Calibration curves illustrated the accuracy of the models obtained by logistic regression in the prediction of high-grade cancer [27]. The model calibration was assessed using the calibration slope and the calibration in the large (CITL) [28].

STATA/MP was used for statistical analysis (StataCorp, College Station, TX, USA); statistical significance was set at *p* < 0.05.

## 5. Conclusions

The models developed to date to refine the indications of prostate biopsies must be reconfirmed and adjusted to the emerging era of upfront MRI and IGB. The present series supported MDx-2 testing in the process of selection after upfront MRI, notably for PI-RADS-v2 score 3 abnormalities. Alternately, relevant complementary information can also be extracted from classical, readily available and inexpensive variables.

## Figures and Tables

**Table 1:** Patient characteristics and biopsy results according to the 2016 World Health Organization classification and the 2014 International Society of Urological Pathology consensus conference on Gleason grading of prostatic carcinoma, and to the technique used to procure biopsies. Medians and 95% confidence intervals are presented for quantitative variables.

Variable	Whole Population (*n* = 117)	No or Low-Grade Cancer (*n* = 85)	High-Grade Cancer (*n* = 32)	p*
Age (years)	65 (63–67)	64 (61–66)	68 (64–70)	0.001
Family history	24/117 (20.5%)	21 (24.4%)	3/30 (10.00%)	0.07
Suspicious DRE	28/117 (23.08%)	19 (22.4%)	9 (28.1%)	n.s. (*p* = 0.51)
Primary prostate Bx	43 (36.75%)	26 (29.89%)	17 (56.67%)	0.024
PSA (ng/mL)	7.0 (6.5–8.0)	6.9 (6.2–8.5)	7.0 (5.5–9.4)	n.s. (*p* = 0.7)
PCA3	27 (23–34)	24 (19–34)	32 (19–52)	n.s. (*p* = 0.24)
Prostate volume (mL)	50 (44–55)	55 (48–62)	38 (33–50)	0.0009
PSA density (ng/mL^2^)	0.15 (0.13–0.18)	0.14 (0.12–0.16)	0.18 (0.12–0.24)	n.s. (*p* = 0.22)
PCPT 2.0 risk estimate (%) Any cancer	28.0 (26.0–30.0)	28.0 (25.0–29.0)	29.5 (25.0–34.0)	n.s. (*p* = 0.24)
PCPT 2.0 risk estimate (%) High-grade cancer	11.0 (9.0–12.0)	11.0 (8.0–12.0)	11.5 (8.0–15.0)	n.s. (*p* = 0.53)
MRI Index Target				
PI-RADS-v2 score 3	29 (24.79%)	26 (30.6%)	3 (9.4%)	
PI-RADS-v2 score 4	64 (54.70%)	47 (55.3%)	17 (53.1%)	0.005
PI-RADS-v2 score 5	24 (20.51%)	12 (14.1%)	12 (37.5%)	
MRI-Biopsy interval (month)	18 (11–25)	22 (14–27)	12 (7–22)	n.s. (*p* = 0.19)
Prostate Biopsy				
Core number				n.s.
Image-guided cores	4 (4–4)	4 (4–4)	4 (4–6)	*p* = 0.52
Systematic cores	10 (9–10)	10 (9–10)	10 (8–10)	*p* = 0.72
Image-guided and systematic cores	14 (14–14)	14 (13–14)	14 (14–15)	*p* = 0.09
Cancer detection				
Image-guided cores	42/117 (35.9%)	12/85 (14.1%)	30/32 (93,8%)	<0.001
Systematic cores	12/117 (10.3%)	10/85 (11.8%)	2/32 (6.3%)	<0.001
Image-guided and systematic cores	54/117 (46.2%)	22/85 (25.9%)	32/32 (100%)	<0.001
SelectMDx-v2 score				
	−2.40 (−2.51/−2.13)	−2.44 (−2.73/−2.25)	−1.89 (−2.42/−1.30)	0.004
<−2.8	36 (30.8%)	31 (36.5%)	5 (15.6%)	0.03
≥−2.8	81 (69.2%)	54 (63.5%)	27 (84.4%)	

Mann–Whitney U-test for quantitative variables or Kruskal–Wallis test for categorical variables. n.s.: not significant.

**Table 2:** Logistic regression of biomarker and clinical models. Odds ratios and regression coefficients are presented for each variable surviving parameter selection after incorporation into the final multivariable model. 95% CI: 95% confidence interval.

Biomarker and Clinical Models	OR [95%CI]	Coefficient [95%CI]	*p*	AUC [95%CI]
Biomarker model				
PI-RADS-v2	2.89 [1.44–5.84]	1.06 [0.36–0.76]	0.03	0.73 [0.64–0.83]
SelectMDx-v2	1.53 [1.06–2.19]	0.43 [0.07–0.79]	0.02	
Clinical model				
PI-RADS-v2	2.49 [1.22–5.08]	0.91 [0.20–1.63]	0.012	0.80 [0.71–0.90]
Age	1.11 [1.02–1.21]	0.11 [0.02–0.20]	0.014	
PSA density	74.39 [0.49–11239]	4.31 [−0.71–9.33]	0.092	
DRE	0.92 [0.30–2.74]	-0.09 [−1.18–1.01]	0.875	

**Table 3:** Performance of PI-RADS-V2 score, SelectMDx-V2 score, biomarker model and clinical model for predicting high-grade cancer on biopsy. * Cut-off value at maximum Youden index. ** As defined by Van Neste and in the SelectMDx-v2 user’s manual.

Predictors	Cut-Off [Range]	Sensitivity	Specificity	Correctly Classified
PI-RADS-v2	4* [3–5]	90.6%	30.6%	47.0%
SelectMDx-v2	−2.8 ** [−4.35 to 4.90]	84.4%	35.3%	48.7%
	−1.99 * [−4.35 to 4.90]	56.3%	75.3%	70.1%
Biomarker model (PI-RADS-v2/SelectMDx-v2)	0.22* [0 to 1]	78.1%	56.5%	62.4%
Clinical model (PI-RADS-v2/Age/PSA/Prostate volume/DRE)	0.24* [0 to 1]	87.5%	68.3%	73.7%
